# German Consumers’ Attitude towards Selenium-Biofortified Apples and Acceptance of Related Nutrition and Health Claims

**DOI:** 10.3390/nu10020190

**Published:** 2018-02-09

**Authors:** Lena Wortmann, Ulrich Enneking, Diemo Daum

**Affiliations:** Osnabrueck University of Applied Sciences, Am Kruempel 31, 49090 Osnabrueck, Germany; u.enneking@hs-osnabrueck.de (U.E.); d.daum@hs-osnabrueck.de (D.D.)

**Keywords:** selenium, apples, nutrition and health claims, functional food, supplements, consumer acceptance

## Abstract

The present study investigates consumers’ acceptance of Se-biofortified apples, as well as Se health and nutrition claims that have been approved by the European Commission. Despite indications that such statements are more likely to be accepted if the carrier product itself has a healthy image, unprocessed fruits biofortified with Se have not been investigated in this context yet. Apples as the most frequently-consumed type of fresh fruit in Germany may offer the potential to improve the Se status of consumers when the produce is enriched with Se. Therefore, an online survey of 356 German adults was conducted to analyze different aspects that could affect the perception of and preference for the proposed product concept by consumers. The findings indicate a moderate acceptance of Se-biofortified apples, as well as of Se health and nutrition claims among the participants. Additional information about beneficial health effects of Se had a significant impact on consumer acceptance. People who regularly eat convenience food and prefer to buy apples at supermarkets were particularly attracted by the product idea. In conclusion, the results of the study indicate good prospects for a successful introduction of Se-rich apples in the German food market, if the produce is advertised with approved health and nutrition claims.

## 1. Introduction

Selenium (Se) is an essential trace element that is important for manifold aspects of human health, including optimal immune response, proper thyroid hormone metabolism, cardiovascular health and prevention of neurodegeneration and cancer [1,2,3]. Se primarily enters the food chain through plants, which take it up from the soil [4,5]. In many parts of Europe, soil Se concentrations are relatively low [6]. Consequently, plant-based food often contributes little to the Se intake of humans. In comparison, food of animal origin may be richer in Se due to the use of feed supplements in livestock production or the application of Se-enriched fertilizers on pastures [7,8]. Hence meat, sausages, eggs, milk and dairy products and, furthermore, fish are usually major food sources of Se in the human diet [9]. Accordingly, a higher Se status in omnivores as compared to vegetarians and vegans was determined in a number of epidemiological studies [10]. Suboptimal Se status was reported to be widespread throughout Europe [11]. The average daily intake of Se in Germany was estimated at 38 μg for women and 47 μg per day for men [12]. According to the reference values of the German Nutrition Society, updated in 2015, optimal Se supply is achieved with a daily Se intake of 60 μg for women and 70 μg for men [13]. The European Food Safety Authority (EFSA) recommends a Se intake of 70 μg per day for adults of both sexes [14].

In areas where soils are low in bioavailable Se, agronomic biofortification has proven to be an efficient and safe way to supply the population with sufficient Se. For example, in Finland, where the Se fertilization has been regulated by the state since 1984, the mandatory supplementation of soil-applied fertilizers with sodium selenate has increased the Se concentration of all major food groups. As a result, the daily dietary intake of Se increased on average from about 40–80 μg per person [15]. In other European countries, the application of Se-enriched fertilizers in food crop production has not been very widespread to date. Although Se has not been classified as an essential element for higher plants, it may improve crop growth, especially under conditions of abiotic and biotic stress [16,17,18]. In addition, positive effects on the quality of vegetables and fruits were reported including higher contents of taste-influencing ingredients (e.g., sugars), as well as of phytochemicals and vitamins (e.g., polyphenols and ascorbic acid). Furthermore, Se may increase the antioxidative capacity and extend the shelf life of plant produce [19,20,21].

The market for functional foods, which are advertised with nutrition and health claims, has an annual turnover of €4.52 billion in Germany [22]. Twenty seven percent of Germans already use such foods once or several times a week. Furthermore, 28% use them at least once a month [23]. Highly rated health attributes are “High in fiber” (36%) and “High in protein” (32%), “Whole grain” (30%), as well as foods that are “Fortified with calcium” (30%), “Vitamins” (30%) or “Minerals” (29%) [24]. Fresh, unprocessed fruit and vegetables do not belong to the regularly-used functional foods in Germany. In addition, previous consumer studies did not investigate the acceptance of fresh unprocessed fruits that offer additional health benefits due to a higher content of Se or other essential trace elements.

It is known that health claims are more likely to be accepted if the carrier product itself is already associated with a healthy food or has a healthy image [25,26]. In this regard, particularly fresh fruits and vegetables offer potential for health claims as different studies suggest that regular or increased consumption of these foods reduces the risk of several chronic diseases [27]. In comparison with other fruits and vegetables, apples appear to be especially suitable for Se biofortification programs in Germany, since they are the most frequently-consumed fruit [28]. Before Se-biofortified apples can be launched in the food market, there is a need for a better understanding of consumers’ perceptions and acceptance of Se-enriched food. This is especially interesting since fresh apples are a healthy, but uncommon basic product for functional foods in Germany. Therefore, the objective of this study was to examine aspects that could promote or reduce the consumer acceptance of Se-biofortified apples. In particular, the aim was to clarify the following research questions:

(1) Is the acceptance of Se-biofortified apples influenced by nutritional behavior, health awareness, usage habits regarding food supplements and demographic aspects?
(2) Which food categories are suitable for optimizing the Se intake from the consumers’ point of view (2a)? Are natural products, such as Se-enriched apples, more likely to be accepted than food supplements (2b)?
(3) Which nutrition (3a) and health claims (3b), approved by the European Commission, are most appealing to consumers with regard to Se-biofortified apples?
(4) Can consumer acceptance of Se-related nutrition claims significantly be improved by providing information regarding the health effects of Se?
(5) How do consumers assess alternative methods to increase the Se content in food?

## 2. Materials and Methods

### 2.1. Study Design

The study was carried out in two phases. In the first stage, we conducted ten qualitative face-to-face interviews, as well as two focus groups. The results obtained were used to adapt and detail the questions and choices of answer where necessary.

The online questionnaire with 356 German consumers in Stage 2 was comprised of the following topics: nutritional behavior, health awareness, usage habits of food supplements and demographics. These dimensions were used to identify potential influencing factors for the concept of Se-biofortified apples (Research Question 1) and are specified below.

To measure how nutritional behavior affects the acceptance of Se-biofortified apples, questions about various nutritional aspects were included. The participants were asked about their usage frequency of convenience food and fresh apples. Respondents could choose one of the following frequencies: never, about once or several times a month, about once or several times a week and daily. Those who consume fresh apples or convenience food more than once a week were defined as “High intensity users”. An additional question relates to the purchase frequency of organic apples in relation to the total fresh apple consumption. Participants were asked if they “always”, “mainly”, “seldom” or “never” buy organically grown apples. The difference in the acceptance of Se-enriched apples between the two top (always, mainly) and bottom (seldom, never) subgroups was analyzed. Furthermore, consumers had to identify the outlets where they usually purchase or obtain apples: discount shops, supermarkets, organic food shops, producers, weekly markets, their own garden, specialist retailers or other sources. Participants were allowed to choose more than one answer.

We hypothesized that the more health-conscious consumers may have more knowledge about essential trace elements such as Se and therefore would tend to accept an innovative product such as Se-biofortified apples. Therefore, some questions about health awareness were included in the survey. These questions relate to how consistently or frequently consumers pay attention to food ingredients and health claims for food. Consumers were asked how often they pay attention to special ingredients when buying foods. Possible answers were: always, mostly, seldom, only if I have enough time, only in specific situations and never. Regarding the health claim awareness, ten typical health claims were presented to the participants. They were asked to select all health claims that had already influenced their purchasing decision for a special food product. Additionally, they were allowed to name further health effects to which they pay attention. Since food supplement users could represent a potential target group for Se-biofortified apples, it was determined whether and how often food supplements are used. Participants could choose one of the following answers: daily, several times a week, less often than that, but regularly (preventive), regularly (in response to complaints), rather irregularly (preventive), irregularly (in response to complaints) and never. Participants were subsequently clustered into two response groups. All those who stated that they used food supplements regularly (preventive or in response to complaints), several times a week or daily are hereinafter defined as the group of supplement users. In addition, participants had to answer whether they believed in the positive effect of the trace element Se. Possible answers ranked from +2 (“Definitely true”) to −2 (“Definitely false”). Moreover, several food categories, such as convenience food, meat and meat products, milk and dairy products, grain and cereal products, vegetables, fruits, pulses, egg products and the option others were presented as possible foods for optimizing the Se status through daily food consumption (Research Questions 1 and 2a). Participants were asked which of those categories they find appealing for the intake of additional Se through daily nutrition. More than one answer could be chosen, and it was possible to select the answer “In principle, I would not consume foods that contain additional Se” as well. At the end of the questionnaire, participants were asked to rate their overall acceptance of the presented concept. This answer was measured on a scale from 1 (“Not appealing at all”) to 7 (“Very appealing”).

The two alternative concepts “Food supplement with Se” versus “Se-rich apple” were evaluated with regard to the aspects: “Healthy”, “Trustworthy”, “Environment-friendly”, “Effective”, ”Expensive”, “Natural”, “Dosage” and “Overall I would rather decide on …” (Research Question 2b).

**Figure A1:**
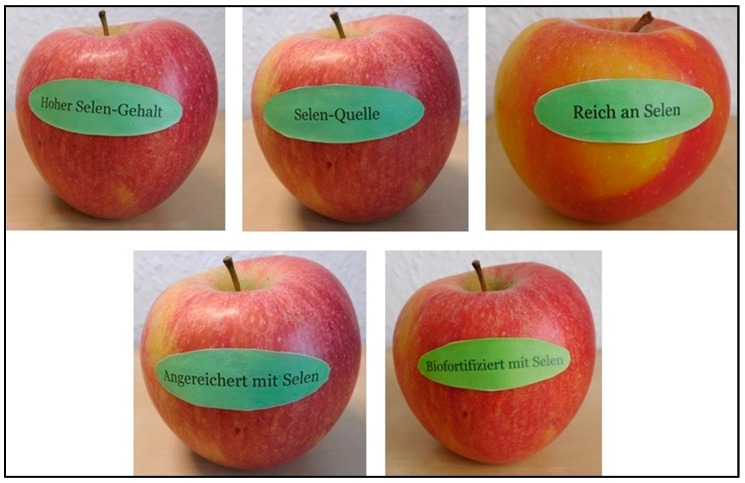
Visual stimulus for Se nutrition claims for apples (translated nutrition claims: High Se content, Source of Se, Rich in Se, Enriched with Se, Biofortified with Se).

To investigate the third research question regarding the acceptance of Se-related health and nutrition claims (Table 1), the participants had to rate the possible claims on how useful or appealing they find these claims. Possible scores ranged from 1 (“Not useful for my health” or “Not appealing at all”) to 7 (“Very useful for my health” or “Very appealing”). To assess the nutrition and health claims, pictures of apples with each claim were presented to the participants for a better simulation of the purchase situation (see Figure A1). In the countries of the European Union, Se-related nutrition and health claims can be used if the food contains a Se concentration of at least 8.25 µg/100 g fresh weight. This is specified by Regulation (EC) No. 1924/2006 on nutrition and health claims made for foods [29], Regulation (EU) No. 1169/2011 on the provision of food information to consumers [30] and Regulation (EU) No. 432/2012 establishing a list of permitted health claims made for foods [31].

**Figure A2:**
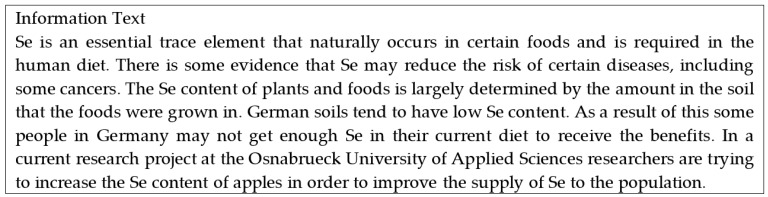
Text box used in the survey to inform participants about the relevance of Se in human nutrition and the need to improve dietary Se intake.

To ascertain whether additional information about Se as an essential part of the human diet has an impact on consumers’ acceptance of the concept of Se-biofortified apples (Research Question 4), a short information box was presented to the survey participants after the first rating of the nutrition claims for Se (see Figure A2). After reading this explanatory text in the box, participants had to rate the nutrition claims again. Possible scores ranged again from 1 (“Not appealing at all”) to 7 (“Very appealing”).

Various methods for increasing the Se content in foods have been presented to the consumers to answer Research Question 5. The methods are listed below:

(1) Application of Se to soils, thereby increasing the Se content in crops
(2) Usage of feed additives containing Se in animal husbandry
(3) Application of Se on plants, especially suitable for fruits and vegetables
(4) Addition of Se during the processing of food
(5) Breeding of certain crop varieties containing Se

These five methods were evaluated by the participants regarding the aspects “Healthy”, “Trustworthy”, “Effective”, “Natural”, “Environment-friendly” and ”Expensive” similar to research Question 2. On a scale from 1–7, a 1 had to be chosen if the characteristic did not apply at all to the specific method from their point of view and a 7 if this aspect totally applied to the method. In addition, the aspects “Taste”, “Apple variety”, “Price”, “Appearance”, “Origin” and “Organic farming” were assessed in terms of how important they are for consumers when purchasing apples. Possible answers ranged from 1 (“Unimportant”) to 7 (“Very important”).

As we hypothesized that the term “Biofortification” is not commonly known from the consumers’ perspective, the wording “Se-biofortified apples” has been replaced by generally understandable formulations (e.g., “Apples with higher Se-content” or “Se-rich apples”) within the consumer questionnaire.

### 2.2. Data Collection and Sample

Five of the qualitative face-to-face interviews of the first stage were conducted in a drugstore market in the department with food supplements in the inner city of Osnabrueck to ensure that consumers who are interested in food supplements were also addressed. The other participants form a sample of randomly-selected persons and students of the University of Applied Sciences Osnabrueck.

In the second stage of the study, 356 persons answered an online questionnaire. These participants were recruited with the help of an online panel (35.7% of the sample), in nutritional magazines and consumer forums (5.2% of the sample) and at the Osnabrueck University of Applied Sciences (59.1% of the sample). The participants were not screened in advance in terms of socio-demographic and psychographic data. Women represented 62.8% of the respondents. This percentage is in line with the common gender distribution in household shopping tasks (65% of women in Germany are solely responsible for food purchasing) [32]. The level of education across the sample is relatively high. Thirty percent of the participants have a university degree compared to 27% in all of Germany [33]. Therefore, we assumed that the status quo with regard to the knowledge and assessment of Se would not be significantly higher in the total population. Sixty one percent of the respondents consume apples several times a week. In addition, 52.5% of the survey participants believe in the positive health effects of Se. Reference is made to Table 2 for a detailed description of the sample characteristics.

### 2.3. Analysis

Statistical analysis was performed using software SPSS v. 24 (IBM Corp., Armonk, NY, USA). For descriptive data analyses, means and standard deviations were determined. Linear regression was applied to explore explanatory factors for the concept of Se-biofortified apples. Two sample *t*-tests were applied to explore if there are gender-specific or other socio-demographic influences on the acceptance of Se nutrition and health claims. One-sample *t*-tests were applied to identify the information effect for the acceptance of Se nutrition claims. Results were considered statistically significant when a two-tailed *p*-value was less than 0.05.

## 3. Results

### 3.1. Influencing Factors on the Acceptance of Se-Biofortified Apples

The acceptance for the concept of Se-biofortified apples of M = 4.19 (SD = 1.65) on a scale from one (“Not appealing at all”) to seven (“Very appealing”) was observed, with 46.6% of respondents reacting positively to the product idea presented. Intensive food supplement users did not significantly prefer this approach in relation to low users and non-users. Furthermore, no statistically-significant difference was found when comparing the two sub-samples of the online panel participants and other participants. A linear regression was performed to find out which independent variables have an influence on how appealing the concept appears to consumers (Table 3).

The results indicate that participants with a high acceptance for Se-biofortified apples generally considered fruit to be the appropriate food category to achieve an optimal intake of Se. Moreover, the acceptance for the product idea was positively associated with a preference for the most favored Se-related nutrition (rich in Se) and health claim (Se contributes to a normal function of the immune system), use of supermarkets as the usual shopping location for apples, belief in the positive health effects of Se and preference for Se-rich apples instead of food supplements containing Se. Participants who consume convenience food more than once a week tended to accept the Se biofortification of apples. Furthermore, acceptance of such functional fruits rose with increasing age. In contrast, consumers who frequently eat apples (more than once a week) were less convinced by the product idea. Likewise, people with Abitur (High School Certificate) or a university degree seem to reject this approach to improve dietary Se intake (Table 3).

Awareness of nutritional contents in food, awareness of health claims for food products, as well as the regular eating of organically-produced apples did not have a significant influence on the acceptance of Se-biofortified apples.

### 3.2. Consumers’ Attitudes towards Different Dietary Se Sources and Influencing Factors in the Decision-Making Process between Se-Biofortified Apples and Food Supplements

**Figure 1:**
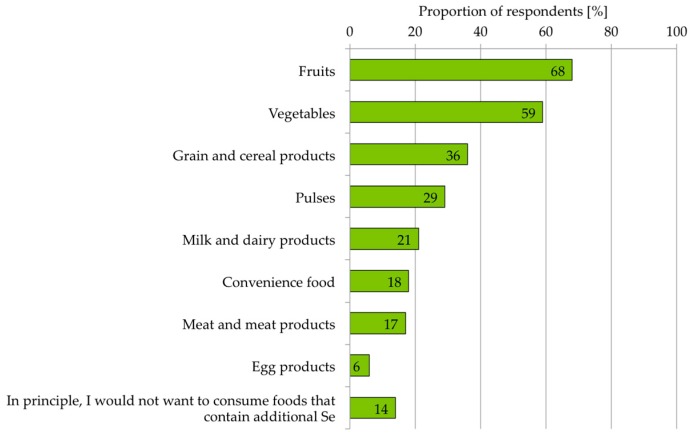
Suitability of different food categories for optimizing the Se intake through daily nutrition according to the participants surveyed. *n* = 291.

In a comparison of different potential food categories for additional Se intake through daily diet, the majority preferred fruits (68%) and vegetables (59%). Thirty six percent and 29% of respondents chose the food categories “Cereals and cereal products” and “Pulses”. Animal foods are less frequently considered to be suitable for taking in additional Se: 21% rated “Milk and dairy products” as suitable, 17% “Meat and meat products” and 6% “Egg products”. Another 14% of respondents generally rejected the idea of taking in additional Se via Se-enriched foods (Figure 1).

**Figure 2:**
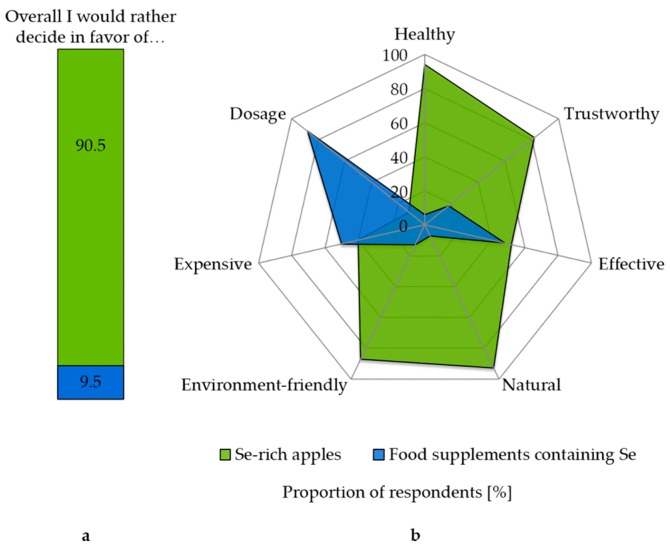
Consumer evaluation of Se-rich apples and food supplements (**a**) and potential decision-making factors (**b**). Study participants were asked: “Which of the following aspects would you rather associate with a Se-rich apple or a food supplement with Se?” Decisions were made between 0 = “Food supplements containing Se” and 1 = “Se-rich apples”. *n* = 284–288.

In a direct comparison with food supplements containing Se, there was a strong tendency to prefer the Se-rich apple. Overall, 90.5% of all survey participants would decide in favor of the Se-enriched apple. Results considering the potential factors influencing the decision-making are shown in Figure 2. Eighty-eight percent of the participants believe that food supplements with Se are easier to consume in the right dosage. However, 60% of respondents expect them to be more expensive. In terms of effectiveness, there is no clear decision for either of the two alternatives, Se-rich apple or food supplements containing Se. In contrast, a clear majority expects the apple to be healthier (94%), more natural (93%), environmentally friendly (87%) and trustworthy (82%).

### 3.3. Acceptance of Nutrition and Health Claims for Se in Apples

**Figure 3:**
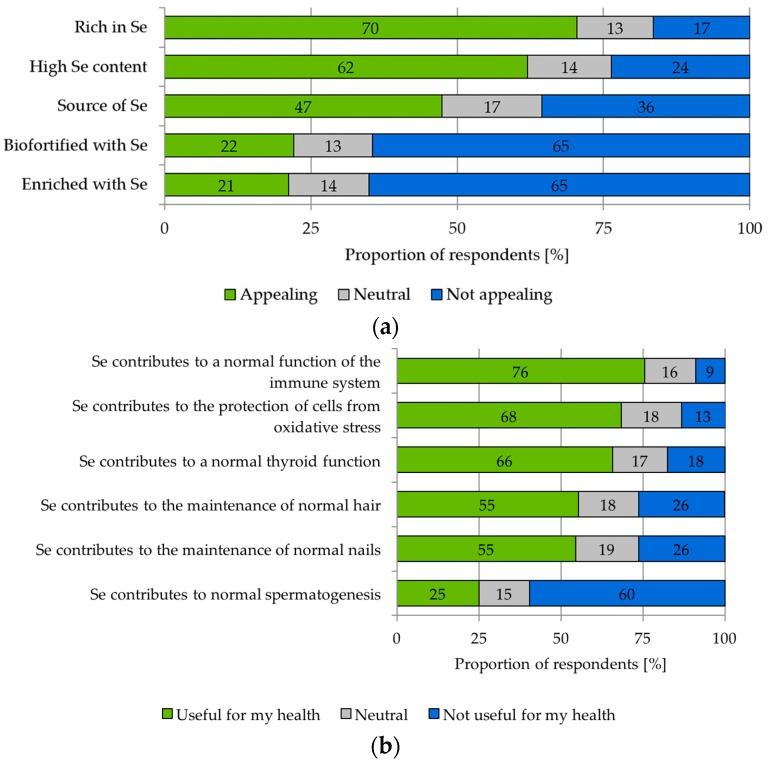
Acceptance of nutrition and health claims for Se-rich apples. Study participants were asked: (**a**) Please rate the following claims for apples with regard to the purchase of these fruits; possible scores ranged from one (“Not appealing at all”) to seen (“Very appealing”); pictures of real apples with a label on them were presented to the participants as stimuli (Figure A1), *n* = 321; (**b**) Please assess whether the following effects of an apple with higher Se content are personally useful to you; possible scores ranged from one (“Not useful for my health”) to seven (“Very useful for my health”), *n* = 312–316.

“Rich in Se” is the most attractive nutrition claim (M = 5.07, SD = 1.73). Seventy percent of the participants considered it appealing. Sixty two percent of the respondents reacted positively towards the description “High Se content” (M = 4.69, SD = 1.85), and 47% of the participants considered “Source of Se” an attractive characteristic (M = 4.19, SD = 1.97). The claims “Biofortified with Se” and “Enriched with Se” did not have any appeal for most of the individuals surveyed (M = 2.95, SD = 1.89 and M = 2.86, SD = 1.81, respectively) (Figure 3a).

With regard to health claims, the results (Figure 3b) demonstrate that the survey participants’ preferred the statement “Se contributes to a normal function of the immune system”. Seventy six percent of them answered that they assess this trait in connection with the fruit apple as useful for their health (M = 5.56, SD = 1.57). Health claims dealing with the protection of cells from oxidative stress (M = 5.17, SD = 1.60) and thyroid function (M = 5.07; SD = 1.82) obtained a similar acceptance level, as well. Health claims relating to hair, nails and spermatogenesis had medium to low mean values (M = 4.43, SD = 1.76; M = 4.41, SD = 1.71 and M = 2.90, SD = 2.10, respectively). *t*-tests showed significant differences between women and men for claims indicating the contribution of Se to maintenance of normal hair (*t*(282) = 2.692, *p* = 0.008, *r* = 0.16), normal nails (*t*(281) = 3.237, *p* = 0.001, *r* = 0.19) and normal spermatogenesis (*t*(279) = −13.708, *p* = 0.000, *r* = 0.63). While women showed a slightly stronger preference for the first two claims, men were distinctly more attracted by the last mentioned claim (women: M = 1.84, SD = 1.55; men: M = 4.58, SD = 1.74).

### 3.4. Effect of Explanatory Information about Se on the Acceptance of Se Nutrition Claims

The results indicate that the possible nutrition claims for Se may be more attractive to consumers if they gain a better understanding of Se and its relevance to human health. All of the five nutrition claims presented received higher rates after reading an explanatory text, even those who were not attracted before (Table 4). *t*-tests demonstrate that there are highly significant positive information effects in all five cases. The strongest impact was found for the claim “High Se content” (*r* = 0.54). Other nutrition claims were less affected by additional information. The slightest effect was detected for the claim “Biofortified with Se” (*r* = 0.29).

### 3.5. Perception of Various Methods for (Bio-)Fortifying Foods with Se

**Figure 4:**
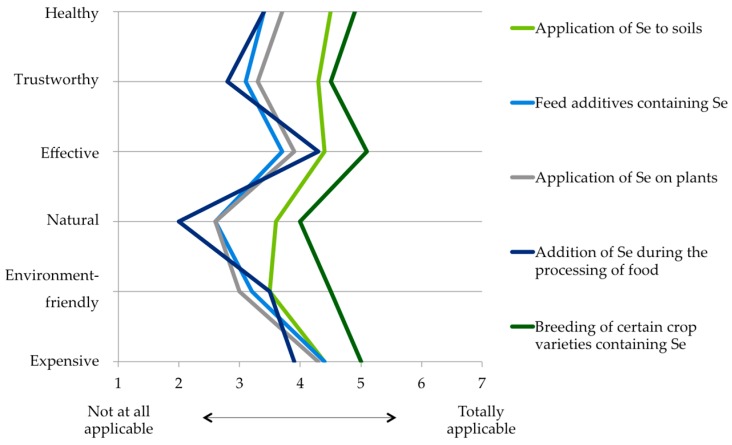
Evaluation of different methods to increase the Se content in food. Study participants were asked: “From your point of view, how strongly do the following characteristics (e.g., “Healthy”) apply to the different methods?” Possible scores ranged from one (“Not at all applicable”) to seven (“Totally applicable”). *n* = 238–258. For all mean values and standard deviations, refer to Table A2.

With reference to different methods to increase the Se content in foods, the participants saw the most appropriate approach in the breeding of crop varieties containing Se, especially with regard to the criteria “Healthy”, “Trustworthy”, “Effective”, “Natural” and “Environment-friendly” (Figure 4). In second place was the fertilization of soils with Se. The lowest consumer acceptance was detected for the addition of Se during food processing. In particular, this method was rated as less “Natural” and “Trustworthy”. Foliar fertilization and the use of feed additives containing Se in livestock farming also received only low acceptance regarding the aspects surveyed.

## 4. Discussion

### 4.1. Se-Biofortified Apples as a Novel Approach to Addressing Se Deficiency in Germany

To improve dietary Se supply in Germany, we suggest that Se-biofortified apples might be an appropriate vehicle from several points of view. Apples are the most important fruit species cultivated in Germany and, furthermore, the most frequently eaten type of fruit. They cover approximately 35% of the annual fruit consumption (23.5 kg of apples per person) [28]. Thus, Se-biofortified apples could reach large consumer groups and would be, due to their good storage suitability, available throughout the year. This availability would allow individuals at risk of Se deficiency to replace Se-containing food supplements with Se-enriched apples. Since vegetarians and vegans are especially affected by a low Se intake [10], apples as non-animal carrier products provide advantages for this consumer group. In areas with conventionally low Se intake, women may develop a severe Se deficit during pregnancy [34]. As a high level of fruit consumption among pregnant women was reported [35], this group of persons could also benefit particularly from the product idea of Se-biofortified apples. Considering the average nutritional Se gaps in Germany of about 20–30 µg Se per person per day [12,13], a Se concentration in apples of about 10–20 µg/100 g fresh weight seems to be a reasonable target range. Food products containing this level of Se can be advertised by using Se-related nutrition and health claims according to relevant EU regulations, e.g., with the statements “Source of Se” and “Se contributes to a normal function of the immune system” [29,30,31]. With the indicated moderate increase in Se concentration, even individuals consuming high amounts of apples will not run the risk of exceeding the tolerable upper intake level for adults of 400 µg Se/day [36].

### 4.2. Influencing Factors on the Acceptance of Se-Biofortified Apples

The given linear regression model for the overall acceptance of Se-biofortified apples explains 48% of the variance. Consumers who regularly eat convenience foods were particularly attracted by this product idea (Table 3). This relationship is also supported by the observation that supermarket buyers preferred the concept of Se-rich apples, since supermarkets allow quick and convenient shopping. Convenience was also identified as an important factor for the acceptance of functional foods in previous studies [37]. In addition, socio-demographic aspects such as age and education level had a significant influence on the acceptance of the concept of Se-rich apples. Regarding age, conflicting statements have been reported in the literature. While some authors argue that a higher degree of food neophobia relates to less willingness to try new functional foods among older people [38], other studies suggest that the elderly are more interested in or have higher awareness of functional foods [25,39] and are more attracted towards Se-enriched foods [40]. The latter hypothesis was supported by our results. However, the validity of this observation is limited by the fact that elderly persons were underrepresented in the online survey that was carried out. A high education level is often positively associated with a high purchase intention and awareness or usage of functional foods [39,41,42,43]. In contrast, we observed an inverse relationship in that respect. Furthermore, surprisingly, health awareness, as well as supplement use habits could not be identified as important factors for the acceptance of Se-biofortified apples. Contrasting findings were reported in several other studies [44,45]. Likewise, Sandmann et al. found a significant impact of health consciousness on consumers’ willingness to use functional foods when investigating the acceptance of vitamin D-fortified products in Germany [46]. Differences in the knowledge about various ingredients may explain the conflicting observations. Vitamin D is probably better known for its importance to human health than Se. Generally, acceptance of functional food is closely associated with beliefs in the effects of and expected benefits from these products [47]. For Se, it can be assumed that this trace element and its relevance for human health are relatively unknown to the general public and even to individuals with higher education [48].

### 4.3. Consumers’ Attitudes towards Different Dietary Se Sources and Influencing Factors in the Decision-Making Process

There are various possibilities for consumers to increase their Se intake such as consuming foods with higher Se content (e.g., Brazil nuts), using Se-enriched food alternatives (functional foods) and taking food supplements containing Se. In Germany and other European countries, food supplements are already in widespread use. According to a current representative study, 16.2% of the German population are supplementing their mineral intake, and 23% of this group of people are including products containing Se in the form of capsules, tablets, pills and other similar forms. Thus, about 4% of the German consumers are using Se-containing food supplements [49]. Brazil nuts are known for their high natural Se content, but are not a commonly-eaten food in Germany. In addition, considerable variations in the Se concentration of Brazil nuts were reported, ranging from 0.03 mg/kg–512 mg/kg fresh weight [9]. In an Australian consumer study, Brazil nuts were evaluated for their acceptance compared to Se-enriched food alternatives. Among bread, breakfast cereals, bakery products, milk products, pasta, meat, Brazil nuts and food supplements, cereal products were the most preferred food for Se-enrichment from consumers’ point of view. Unfortunately, fruits and vegetables were not included in this context [40]. 

Based on the present results, we conclude that apples as a carrier product for Se are clearly preferred over food supplements. In a direct comparison, apples were more strongly associated with the characteristics naturalness, health, trustworthiness and environmental friendliness (Figure 2). Furthermore, the participants surveyed consider fruits and vegetables in general as the most suitable food categories for this purpose (Figure 1). Interestingly, food of animal origin, such as meat, eggs and milk, was regarded as less appropriate for improving Se intake, although these products supply most of the dietary Se to date [9]. This indicates that only a small group of people is aware of current Se sources.

### 4.4. Acceptance of Nutrition and Health Claims for Se in Apples

Moderate acceptance was detected for several nutrition and health claims related to Se-biofortified apples. Considering that in the present study, both the carrier product and ingredient are unusual for the consumer in connection with functional food, unexpected positive reactions towards most of the claims were obtained. Most of the participants assessed the claim “Se contributes to normal function of the immune system” as appealing (Figure 3b). Similar results were found in a consumer survey that investigated the acceptance of functional foods in Germany more generally. 56% of the participants in this study would try a product that “strengthens the immune system” [50]. In our study, the acceptance level was even higher at 76%. Obviously, this trait and apples fit together very well in the view of consumers. Health claims are reportedly more likely to be accepted if the carrier product itself is already associated with healthy food or has a healthy image [25,26].

With regard to nutrition claims for apples, the expression “Rich in Se” was significantly preferred to the expression “Enriched with Se” (Figure 3a). The low acceptance for the latter description is probably due to the fact that the participants regard such a product as less natural. Participants in our preceding qualitative survey had already expressed concerns about the naturalness of apples enriched with Se. Accordingly, when evaluating different methods to increase the Se content in food, respondents assess the “Addition of Se during food processing” as the least natural and trustworthy approach (Figure 4). Previous studies have also identified consumers’ potential trade-offs between the healthiness and naturalness of functional food, and perceived naturalness is considered as an important attribute in this product segment [51,52,53,54]. In this light, fresh produce such as apples might be more favorable than processed food products.

A conscious and strategic use of health and nutrition claims might not only be essential for successful marketing of Se-biofortified apples, but possibly also offers potential to promote educational effects. Ippolito and Mathios point out that health claims lead to increased consumer knowledge about the relationship between the intake of certain nutrients and the improvement of health status or risk reduction in the development of certain diseases [55].

### 4.5. Effect of Information about Se on the Acceptance of Se Nutrition Claims and Strategies to Launch Se-Biofortified Products in the Food Market

Several studies demonstrate that the acceptance of functional ingredients increases when they are on the market for some time and therefore in the mind of consumers [56,57]. As Se is rather unknown as an antioxidant and essential trace element [40] and not yet established in functional food in Germany, we provided participants at a later stage of the survey with some background information about the relevance of Se for humans. As hypothesized, the acceptance of nutrition claims increased after information on the health benefits of adequate Se intake had been given.

As already mentioned by Cox et al., there is at first a need to promote Se and foods enriched with this trace element to overcome knowledge deficits in the relevant population [40]. To point out the advantages of Se-biofortified apples for consumers, different possibilities for retailers are available, like package design, labeling and branding. If Se-biofortified apples are introduced as a premium product with a reasonable price increase as compared to common produce, all actors along the agri-food chain could benefit from this concept, similarly to other recent innovations in the fruit market (e.g., apple club varieties such as Pink Lady^®^ and Kanzi^®^). With regard to the commercialization of Se-biofortified fruits, interesting side effects of Se on marketable quality should be taken into account. For example, Pezzarossa et al. reported that foliar sprays with sodium significantly increased the soluble solids content in pears and flesh firmness in peaches [20]. The flavor and taste of fruits are closely related to their level of soluble solids, which are mainly sugars. Taste was identified in our study as the most important decision factor when buying apples (Table 2). This is in line with other reports [51]. A delayed reduction in flesh firmness positively affects the shelf-life of fruits. Se may also stimulate the formation of ascorbic acid and polyphenolics in fruits [21,58,59]. These bioactive substances substantially contribute to the health value of fruits [27].

### 4.6. Limitations of the Study Presented and Implications for Further Research

Due to the completely novel combination of fresh unprocessed apples biofortified with Se, this study aimed first of all to investigate different aspects affecting consumers’ attitude towards this functional fruit with regard to its market introduction and commercialization. Further research should focus more on psychographic aspects and personal motives for using or avoiding fresh foods enriched with Se. The sample size used in our study was deemed to be sufficient to investigate the research questions posed, but not representative of the German population. Therefore, conclusions cannot be drawn without reservations for Germany as a whole. Nevertheless, since the educational level was relatively high within the sample (30.0% have a university degree compared to 27% across Germany [33], it can be assumed that the status quo regarding the knowledge and assessment of Se will be quite similar throughout the German inhabitants. However, there is still a need for a representative study in order to prove this. Further limitations relate to the cultural context: As only participants living in Germany were included in the survey, no generalization could be made for populations in other countries, especially for those where dietary habits and attitudes towards functional foods are different. To clarify such relationships, comparative analysis in several countries would be enlightening. In addition, contextual aspects in assessing the Se-biofortified apples have not been considered so far. Since these factors also play an important role in the purchasing decision, it would be interesting to integrate Se-biofortified apples into realistic settings, e.g., supermarkets. Under these test conditions a choice experiment could be conducted by offering the fruits with different nutrition and health claims, as well as with regard to further influencing factors such as price and packaging.

## 5. Conclusions

Apples appear to be a suitable carrier for Se biofortification, since they are generally considered to be a healthy fruit, and several of the Se health and nutrition claims received sufficiently high acceptance rates by the surveyed participants. With regard to Se itself, it is obvious that consumers prefer an apple that obtains the attribute “Rich in Se” as naturally as possible. Approaches considered as artificial or claims implying this, such as the statement “Enriched with Se”, may impair the acceptance of this produce. Sensorial fruit attributes (e.g., taste, flesh firmness, peel color) are known as key purchasing criteria for apples. If these and further quality traits could be positively influenced by Se, as indicated in previous investigations on other fruit species, this would facilitate the entry of the Se-biofortified apples into the food market. However, before this, it is necessary to develop a reliable and efficient method to biofortify apples with Se, which easily fits in with commercial fruit production. Furthermore, it should be studied to what extent Se supplied from apple fruits is utilized in the human body and thus actually may contribute to alleviating Se deficiency in at-risk population groups such as vegans and vegetarians.

## Figures and Tables

**Table 1:** Se-related nutrition and health claims the consumers were asked about.

Nutrition Claims	Health Claims
Rich in Se High Se content Source of Se Biofortified with Se Enriched with Se	Se contributes to a normal function of the immune system Se contributes to the protection of cells from oxidative stress Se contributes to the normal thyroid function Se contributes to the maintenance of normal hair Se contributes to the maintenance of normal nails Se contributes to normal spermatogenesis

**Table 2:** Characteristics of the consumer sample (for additional information concerning important purchasing criteria for apples, refer Table A1).

Variable	Description of Respondents
Gender	37.2% male; 62.8% female
Age	18–70 years of age (M = 30 years of age; SD = 10.82)
Children per household (<18)	18.5% have children living in the household
Educational level	56.2% have Abitur (High School Certificate), 30.0% have a university degree
Method of participation	35.7% online panel, 64.3% other participants
Use of convenience food	26.4% are intensive users of convenience food
Consumption of raw apples	61.0% are intensive users of raw apples
Shopping location	83.5% buy apples in supermarket
Use of organically produced apples	33.9% buy always or mainly organically-produced apples
Awareness of nutritional contents in food	10.5% have a high awareness of nutritional contents
Awareness of health claims	16.6% with high awareness of health claims
Use of food supplements	21.6% are regular users of food supplements
Positive health effects of Se	52.5% believe in the positive health effects of Se
Purchasing criteria for apples	Taste was with M = 6.60 (SD = 0.917) the most important criterion

M = mean value, SD = standard deviation.

**Table 3:** Factors affecting the acceptance of the concept for Se-biofortified apples.

Predictor	Regression Coefficient B	Standard Error	Standardized Coefficient Beta	*t*	*p*
Appealing food category for Se intake (fruit)	1.120	0.176	0.318	6.344	0.000
Acceptance of Se nutrition claims (rich in Se)	0.266	0.049	0.275	5.441	0.000
Acceptance of Se health claims (Se contributes to a normal function of the immune system)	0.218	0.056	0.201	3.882	0.000
Usual shopping location for apples (supermarket)	0.553	0.210	0.129	2.634	0.009
Belief in the positive effect of Se as a micronutrient	0.233	0.084	0.128	2.787	0.006
Preference for Se-rich apples vs. supplements	0.635	0.264	0.114	2.401	0.017
Consumption of convenience food more than once a week	0.383	0.167	0.105	2.289	0.023
Age	0.016	0.008	0.102	2.034	0.043
Awareness of nutritional contents in food (answer: always)	0.463	0.251	0.085	1.841	0.067
Awareness of health claims (number of chosen claims out of 10 possible health claims)	0.042	0.026	0.079	1.635	0.103
Usual shopping location for apples (specialist retail)	0.564	0.330	0.078	1.710	0.089
Consumption of organically-produced apples (top 2 sub-groups: always and mainly)	0.243	0.166	0.071	1.468	0.143
Consumption of raw apples more than once a week	−0.362	0.157	−0.108	−2.315	0.021
Abitur (High School Certificate) or university degree	−0.520	0.224	−0.113	−0.319	0.021
Constant	0.074	0.603		−0.122	0.903

*F* (14) = 18.291; *p* = 0.000; *R*^2^ = 0.508; adjusted *R*^2^ = 0.480. *n* = 263.

**Table 4:** Effect of explanatory information on consumer acceptance of Se-related nutrition claims used for apples.

Claim	Before Information Text	After Information Text	Test of Difference
Rich in Se			
M:	4.57	5.07	*t*(d.f. = 320) = −7.186
SD:	1.84	1.73	*p* = 0.000 (hs); *r* = 0.37
High Se content			
M:	3.71	4.69	*t*(d.f. = 320) = −11.508
SD:	1.89	1.85	*p* = 0.000 (hs); *r* = 0.54
Source of Se			
M:	3.57	4.19	*t*(d.f. = 320) = −8.889
SD:	1.92	1.97	*p* = 0.000 (hs); *r* = 0.44
Biofortified with Se			
M:	2.56	2.95	*t*(d.f. = 320) = −5.413
SD:	1.76	1.89	*p* = 0.000 (hs); *r* = 0.29
Enriched with Se			
M:	2.16	2.86	*t*(d.f. = 320) = −9.265
SD:	1.53	1.81	*p* = 0.000 (hs); *r* = 0.45

*n* = 321. M = mean value, SD = standard deviation, d.f. = degrees of freedom, *p* = significance level, hs = highly significant. Acceptance of the Se-related nutrition claims for apples was measured on a scale from 1 (“Not appealing at all”) to 7 (“Very appealing”).

## Appendix A

**Table A1:** Importance of purchasing criteria for apples.

Criteria	M	SD
Taste	6.60	0.92
Origin	5.09	1.73
Appearance	4.63	1.50
Price	4.28	1.49
Apple variety	4.22	1.90
Organic farming	3.82	1.82

M = mean value, SD = standard deviation; participants were asked: “Which of the following criteria are important to you when purchasing apples?” Possible answers ranged from 1 (“Unimportant”) to 7 (“Very important”). *n* = 290–291.

**Table A2:** Evaluation of different methods to increase the Se content in food.

	Application of Se to Soils	Feed Additives Containing Se	Application of Se on Plants	Addition of Se During the Processing of Food	Breeding of Certain Crop Varieties Containing Se
Healthy					
M:	4.50	3.36	3.66	3.38	4.84
SD:	1.86	1.66	1.69	1.73	1.86
Trustworthy					
M:	4.25	3.06	3.34	2.81	4.52
SD:	1.89	1.71	1.61	1.55	1.93
Effective					
M:	4.42	3.66	3.88	4.32	5.10
SD:	1.65	1.64	1.65	1.80	1.60
Natural					
M:	3.56	2.62	2.65	1.99	4.04
SD:	2.13	1.66	1.62	1.37	2.06
Environment-friendly					
M:	3.50	3.23	3.04	3.51	4.52
SD:	1.87	1.73	1.54	1.75	1.94
Expensive					
M:	4.43	4.36	4.32	3.92	4.99
SD:	1.69	1.59	1.56	1.61	1.69

M = mean value, SD = standard deviation; Study participants were asked: “From your point of view, how strongly do the following characteristics (e.g., “Healthy”) apply to the different methods?” Possible scores ranged from 1 (“Not at all applicable”) to 7 (“Totally applicable”). *n* = 238–258.
